# Targeting α-Synuclein in Parkinson's Disease by Induced Pluripotent Stem Cell Models

**DOI:** 10.3389/fneur.2021.786835

**Published:** 2022-01-25

**Authors:** Angeliki Spathopoulou, Frank Edenhofer, Lisa Fellner

**Affiliations:** Department of Genomics, Stem Cell Biology and Regenerative Medicine, Institute of Molecular Biology and Center for Molecular Biosciences Innsbruck, Leopold-Franzens-University Innsbruck, Innsbruck, Austria

**Keywords:** induced pluripotent stem cells, α-synuclein, Parkinson's disease, neurons, *SNCA* triplication, *SNCA* duplication, A53T *SNCA* point mutation

## Abstract

Parkinson's disease (PD) is a progressive, neurodegenerative disorder characterized by motor and non-motor symptoms. To date, no specific treatment to halt disease progression is available, only medication to alleviate symptoms can be prescribed. The main pathological hallmark of PD is the development of neuronal inclusions, positive for α-synuclein (α-syn), which are termed Lewy bodies (LBs) or Lewy neurites. However, the cause of the inclusion formation and the loss of neurons remain largely elusive. Various genetic determinants were reported to be involved in PD etiology, including *SNCA, DJ-1, PRKN, PINK1*, LRRK2, and *GBA*. Comprehensive insights into pathophysiology of PD critically depend on appropriate models. However, conventional model organisms fall short to faithfully recapitulate some features of this complex disease and as a matter-of-fact access to physiological tissue is limiting. The development of disease models replicating PD that are close to human physiology and dynamic enough to analyze the underlying molecular mechanisms of disease initiation and progression, as well as the generation of new treatment options, is an important and overdue step. Recently, the establishment of induced pluripotent stem cell (iPSC)-derived neural models, particularly from genetic PD-variants, developed into a promising strategy to investigate the molecular mechanisms regarding formation of inclusions and neurodegeneration. As these iPSC-derived neurons can be generated from accessible biopsied samples of PD patients, they carry pathological alterations and enable the possibility to analyze the differences compared to healthy neurons. This review focuses on iPSC models carrying genetic PD-variants of α-syn that will be especially helpful in elucidating the pathophysiological mechanisms of PD. Furthermore, we discuss how iPSC models can be instrumental in identifying cellular targets, potentially leading to the development of new therapeutic treatments. We will outline the enormous potential, but also discuss the limitations of iPSC-based α-syn models.

## Introduction

Parkinson's disease (PD) is the second most common progressive neurodegenerative disorder worldwide, affecting 0.3% of the global population (1), with the majority of the cases manifesting among patients over 70 years of age (2). The main neuropathological hallmarks of PD are the loss of dopaminergic (DA) neurons in the pars compacta of the substantia nigra (SNpc) and DA terminals in the striatum (3), along with the occurrence of neuronal (4, 5) and glial (6) cytoplasmic aggregations of the misfolded protein α-synuclein (α-syn). The neuronal α-syn inclusions prevail compared to glial inclusions in PD and they are termed as Lewy bodies (LBs) and Lewy neurites (LNs) (7). PD belongs to the so-called α-synucleinopathies (ASP), also including dementia with Lewy bodies (DLB) and multiple system atrophy (MSA) [reviewed in Arnaoutoglou et al. (8) and Krismer and Wenning (9)]. In the current review, we will discuss studies employing iPSC-derived PD models, focusing especially on *SNCA* mutations. We will explain the generation of human neuronal cell and organoid models and furthermore discuss the advantages as well as the limitations of iPSC-derived human models.

### Features and Pathology of Parkinson's Disease

The first clinical observations of the disorder were reported by James Parkinson in 1817, describing the disease as “shaking palsy,” a combination of the loss of muscular power of the limbs and their involuntarily shaking (10). Nowadays, it is well-established that the disease is characterized by a combination of both motor and non-motor symptoms. The cardinal motor symptoms are tremor, rigidity, postural instability and bradykinesia (11–16) while the non-motor features include sleep disturbances (10, 17), cognitive and neuropsychiatric abnormalities (18, 19) and dysfunctions of the autonomic nervous system (20, 21). Diagnosis of PD is challenging, especially in its early stage, as it is based on clinical symptoms only.

The degeneration of the SNpc DA neurons together with the loss of DA neuronal terminals in the striatum can lead to a decrease of dopamine of around 80%, resulting in the prominent motor symptoms typical for PD (22, 23) [for a more thorough characterization on midbrain DA neurons consult (24, 25)]. Though the exact mechanisms leading to PD still remain elusive, recent studies indicate the involvement of processes such as oxidative stress, mitochondrial dysfunction, dysregulated autophagic and proteasomal degradation of α-syn, as well as neuroinflammation (26–30). Especially, neuroinflammation driven by over-activated glial cells is considered to play a crucial role in the loss of DA cells (31). Studies using PD animal models and human post-mortem brain tissue suggest the involvement of microglial and also astroglial neuroinflammatory responses that contribute to the disease initiation and progression (32–34). Other studies proposed neuronal loss in PD to be caused by the development of α-syn aggregates following a prion-like mechanism (35–40). Kordower et al. and Li et al. report LB propagation from the PD host to grafted neurons in patients after transplantation (41, 42), indicating a prion-like, “infectious” propagation of α-syn. However, Hallet et al. report healthy and unaffected morphology of DA neurons after transplantation (43). Moreover, the prion hypothesis fails to explain the pathological findings of many post-mortem PD brain specimens, which show distinct areas where different cell types are not equally affected, indicating a selective mechanism behind the pathology (44–46). Recently, a cortical pathogenic theory was proposed by Foffani and Obeso (47). According to this hypothesis the corticostriatal activity could act as a stressor on the nigrostriatal terminals, leading to neuronal degeneration and eventually to PD onset (47). Another hypothesis suggests the involvement of the gut-brain axis in PD onset and propagation, revealing consistent alterations between the microbiome of healthy and PD individuals (48–52). Despite studies supporting all the different hypotheses described above, none of the aforementioned theories is able to explain all pathophysiological PD hallmarks.

PD is classified into idiopathic and familial forms of the disease. The cause of idiopathic PD remains unknown, even though it is assumed that it is caused by a combination of genetic and environmental factors. Several studies link exposure to different pesticides, like rotenone and paraquat (53) or the chemical compound 1-methyl-4-phenyl-1,2,3,6-tetrahydropyridine (54, 55) to the onset of PD. Moreover, other parameters like the patients' diet (56), metabolism (57), inflammation (58) and exercise (59) has been suggested to be involved in the manifestation of PD.

### The Pathophysiological Role of α-Synuclein

Familial PD is linked to genetic variants of PD-related risk genes, following an autosomal dominant or recessive inheritance pattern. One of the first genes identified to be connected with PD pathology was the α-syn gene (*SNCA*) (4). Other PD-relevant genetic mutations include variants of DJ-1, LRRK2 and PINK1 amongst others [for details see (60)]. The present review will focus on the *SNCA* genetic variants. The genetic PD-related risk variants of α-syn are characterized at protein level by point mutations in the N-terminal α-helices, like A30P (61, 62), A53T (63), H50Q (64), and E46K (65). In these cases, it has been reported that the mutant α-syn favors a faster formation of fibrils, which are thought to be more toxic, compared to the wild-type protein (66–69). Moreover, duplications (70) and triplications, respectively, of the *SNCA* gene (71) have been described in PD patients. These multiplications lead to increased levels of α-syn that subsequently result in the emergence of the disease pathology (71–73).

**Figure 1:**
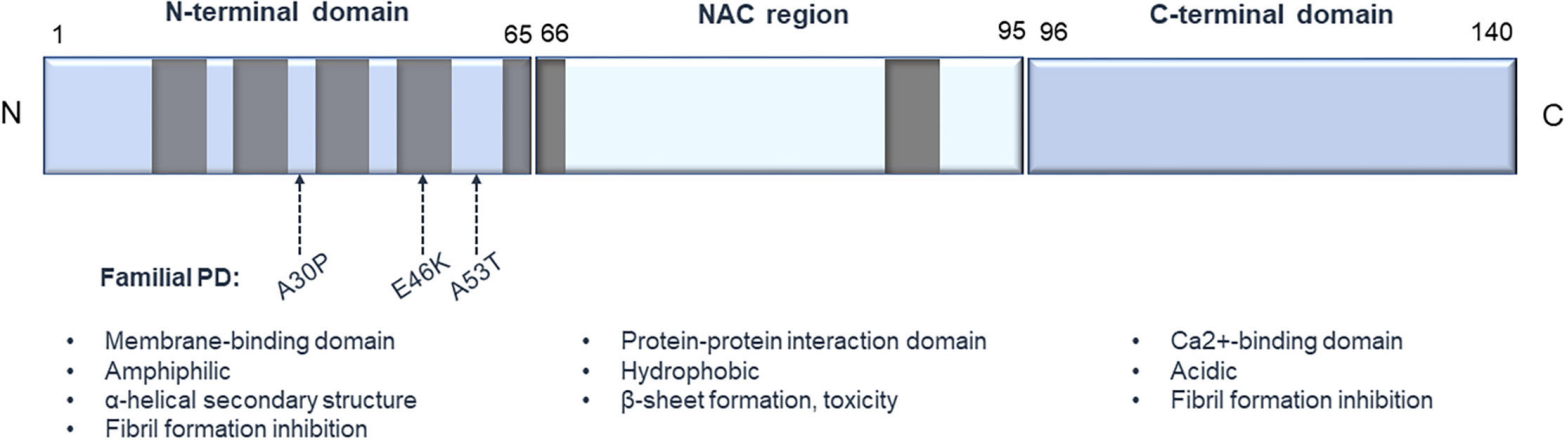
Structure of human α-synuclein. The protein consists of 3 main structural elements: (1) an amphiphilic N-terminus characterized by KTKEGV conserved motifs, important for binding to membranes; (2) an internal hydrophobic NAC domain that is involved in α-syn aggregation; and (3) an acidic C-terminus, which is important for Ca^2+^ binding. Gray boxes represent the KTKEGV motifs, and the arrows designate some of the common point mutations that are connected to PD pathology.

Mutations of the α-syn gene as well as different forms of α-syn, like protofibrils and oligomers, are considered as key players in PD pathogenesis (74, 75). α-syn, also known as the precursor protein of non-amyloid beta/A4 protein (NACP), is encoded by the *SNCA* gene and is a member of the synuclein protein family, together with β-synuclein (β-syn), γ-synuclein (γ-syn), also known as synoretin (76–78). At the protein level, human α-syn is 140 amino acids in length and consists of 3 structural elements, a highly conserved amphiphilic N-terminus characterized by KTKEGV motif repetitions (76, 79), the non-amyloid beta component (NAC) region, which is a hydrophobic internal region and an acidic C-terminus (80) (Figure 1). *In vitro* the wild-type protein does not show a fixed secondary conformation (81–84). However, the protein has the capacity to form fibrillar aggregates both *in vitro* and *in vivo*. The N-terminal domain favors an α-helical conformation, which is expected to hinder the appearance of the toxic, fibril-related β-sheet formations of the NAC region (85–87). Truncated C-terminal α-syn variants are found to be prone to fibrilization both *in vitro* (88–90) and *in vivo* (91–94) indicating a potential fibril inhibitory role of the C-terminal domain. α-syn aggregations can also be enhanced by a variety of other parameters, like phosphorylation (95), nitration and oxidation (96, 97), ionic strength (98) or acidic pH (99).

The physiological role of the α-syn protein is still unclear. It has been reported that α-syn is involved in the synaptic regulation and maturation (74, 100, 101). Moreover, α-syn knockout mice exhibit a DA reduction in the striatum, indicating a potential role of α-syn in DA neurotransmission (101). Decreased synaptic levels of α-syn in primary neurons led to a decrease in the levels of presynaptic proteins, like synaptophysin and synapsin I, that are important for vesicle formation (102). The protein appears to also be implicated in vesicle endocytosis and degradation (103–105). It has been shown that α-syn interacts with mitochondrial and lysosomal membranes (106, 107). Elevated levels of α-syn has been reported to lead to increased mitochondrial fragmentation in ESC-derived neurons (108). Several studies also reported the influence of α-syn in mitochondrial fusion/ fission (109, 110). The protein has also been detected in the nucleus, with recent studies reporting the effect of nuclear α-syn on transcriptional regulation (111, 112) and epigenetic modifications (113). It is apparent that despite the numerous studies in the field, the actual physiological role of α-syn remains elusive.

### Generation of Midbrain DA Cells From iPSCs

Recently, induced pluripotent stem cells (iPSCs) emerged as novel tools to study human pathology in general and PD in particular. Human iPSCs were first reported by *in vitro* reprogramming of adult human fibroblasts, as published by Takahashi et al. (114). Like embryonic stem cells (ESCs), iPSCs are pluripotent stem cells that are able to self-renew indefinitely and differentiate into the three germ layers, endoderm, mesoderm and ectoderm (114, 115). However, in contrast to ESCs the origin of iPSCs is not the embryonic blastocyst, hence their use bypasses a major bioethical concern associated with the use of human ESCs (116). The major advantage of iPSC-derived models is that they allow the recapitulation of the patient-specific genetic background, offering a model of human origin. iPSCs are also eligible for the generation of isogenic control lines with the use of genome editing tools, like CRISPR (clustered regularly interspaced short palindromic repeats)/Cas9 (117, 118). Since iPSCs can be differentiated into any cell type of interest, they allow the study of complex human disorders where patients' material is difficult to access, particularly for pathologies affecting the human brain (119–121).

The generation of iPSC-derived midbrain DA neurons is highly desired as a meaningful *in vitro* PD model. A human iPSC-DA model could help to study α-syn-driven neurodegeneration which is predominantly occurring in DA neurons in the SNpc in PD. Prior to the establishment of the iPSC technology, ESCs were the main source of DA neurons of human origin to study DA function and get transplantable material. The evolvement and refinement of DA differentiation methods resulted in protocols that can be classified into three main techniques, feeder-dependent methods (122), two-dimensional (2D) monolayer cultures with dual SMAD inhibition [with the use of the small molecules Noggin and SB431542 (123)], and methods that generate embryoid bodies (EBs) (124–126). First differentiation attempts involved the co-culture of ESCs with stromal feeder cells that induce ESC neuronal DA differentiation (122). However, co-culture of ESCs with feeder cells is a rather undefined process as it is difficult to recapitulate how much, and which factors the feeder cells release into the medium. Lee et al. first described a feeder-free, EB-based differentiation protocol. EBs are aggregates developing from self-assembly of iPSC that undergo gastrulation-like processes. Lee and coworkers initially obtained ~7% tyrosine hydroxylase positive (TH+) neurons, a marker indicating DA generation. After including sonic hedgehog (SHH), ascorbic acid (AA) and fibroblast growth factor 8 (FGF8) into the media, they were able to increase the yield of TH+ neurons to ~34% (124). Kawasaki et al. described a feeder-dependent DA differentiation protocol, that leads to a similar TH+ yield but is significantly simpler compared to the protocol from Lee et al. Using PA6 stromal cells they report emergence of ~30% TH+ DA neurons in a neuron-specific class III beta-tubulin (TuJ)-positive neuronal population, which comprises around 52% of total cells (122). Moreover, the differentiated neurons have been shown to integrate into the striatum of mice after implantation. Still, these co-culture systems resulted in highly heterogeneous neuronal populations, including GABAergic, cholinergic, and serotonergic neurons in TuJ-positive neuronal population, resulting in low percentages of DA neurons (122).

**Figure 2:**
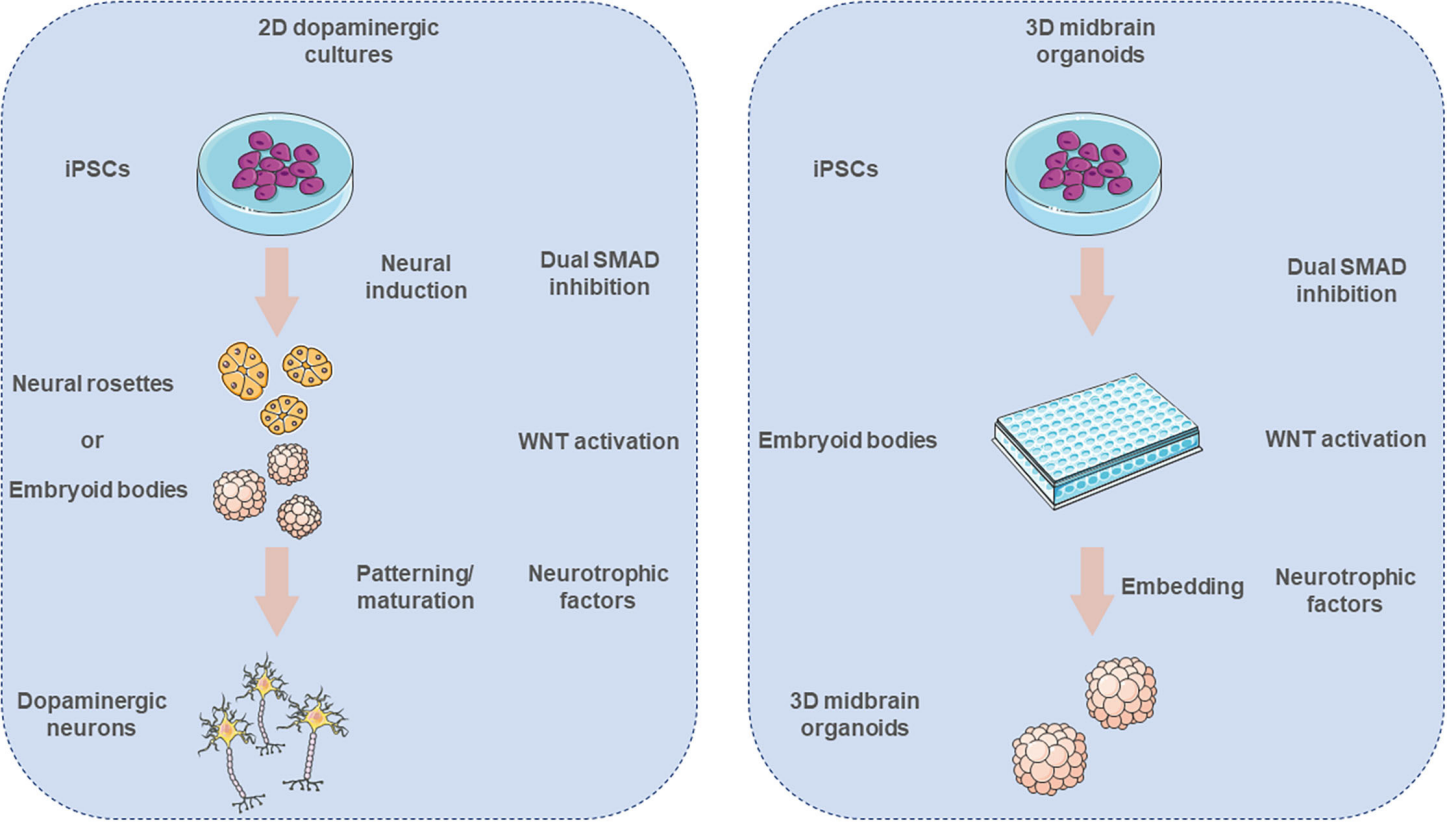
Derivation of *in vitro* iPSC-derived 2D dopaminergic neuronal cell cultures and 3D midbrain organoids. Neural induction of iPSCs is promoted *via* dual SMAD inhibition. Pluripotent cells form neural rosettes or embryoid bodies (EBs), that subsequently are patterned toward ventral midbrain identity. After the initial patterning the neurons are further cultured with chemically defined media in order to give rise to mature midbrain neuronal cultures. In the case of the midbrain organoid cultures, first the iPSCs are forming EBs and then get embedded into an extracellular matrix that supports the 3D growth of these organoids. The illustration was created using images from https://smart.servier.com/.

The increasing need for obtaining more homogeneous DA neuronal cultures was met by exposing the iPSCs in chemically defined media compositions that mimic the physiological developmental cues. One of the first approaches, developed in 2009 by Chambers et al., comprised the differentiation of iPSCs by blocking SMAD signaling with the use of two small molecule inhibitors, Noggin and SB31542 (123). A year later Fasano et al. described the derivation of floor plate (FP) origin DA neurons and proposed an early high dose SHH exposure resulting in the generation of forkhead box A2 positive (FOXA2+) cells (127). The combination of early SHH activation along with the activation of the canonical Wnt pathway, through the use of CHIR99021, a glycogen synthase kinase 3 (GSK3B) inhibitor, led to the generation of neuronal cells that were expressing various FP markers, like TH, FOXA2, LIM homeobox transcription factor 1 alpha (LMX1A), nuclear receptor NURR1 (also known as NR4A2), and paired like homeodomain 3 (PITX3) (128). Further improvements were reported by the additional supplementation of BMP5/7 that robustly increases the *in vitro* differentiation of human iPSCs to midbrain DA neurons up to 3-fold (129). By using further optimized DA differentiation protocols the groups of Parmar (126), Takahashi (130, 131), Barker (132), and Studer (123) were able to provide preclinical evidence that hESC-derived DA neurons are functionally equivalent to those derived from fetal tissue, supporting continued development of hESC-derived cells as a clinical approach for cell replacement treatment of PD (133). The main strategy for the generation of iPSC-derived midbrain DA neurons is illustrated in Figure 2.

### Generation of Midbrain Organoids From iPSCs

**Figure 3:**
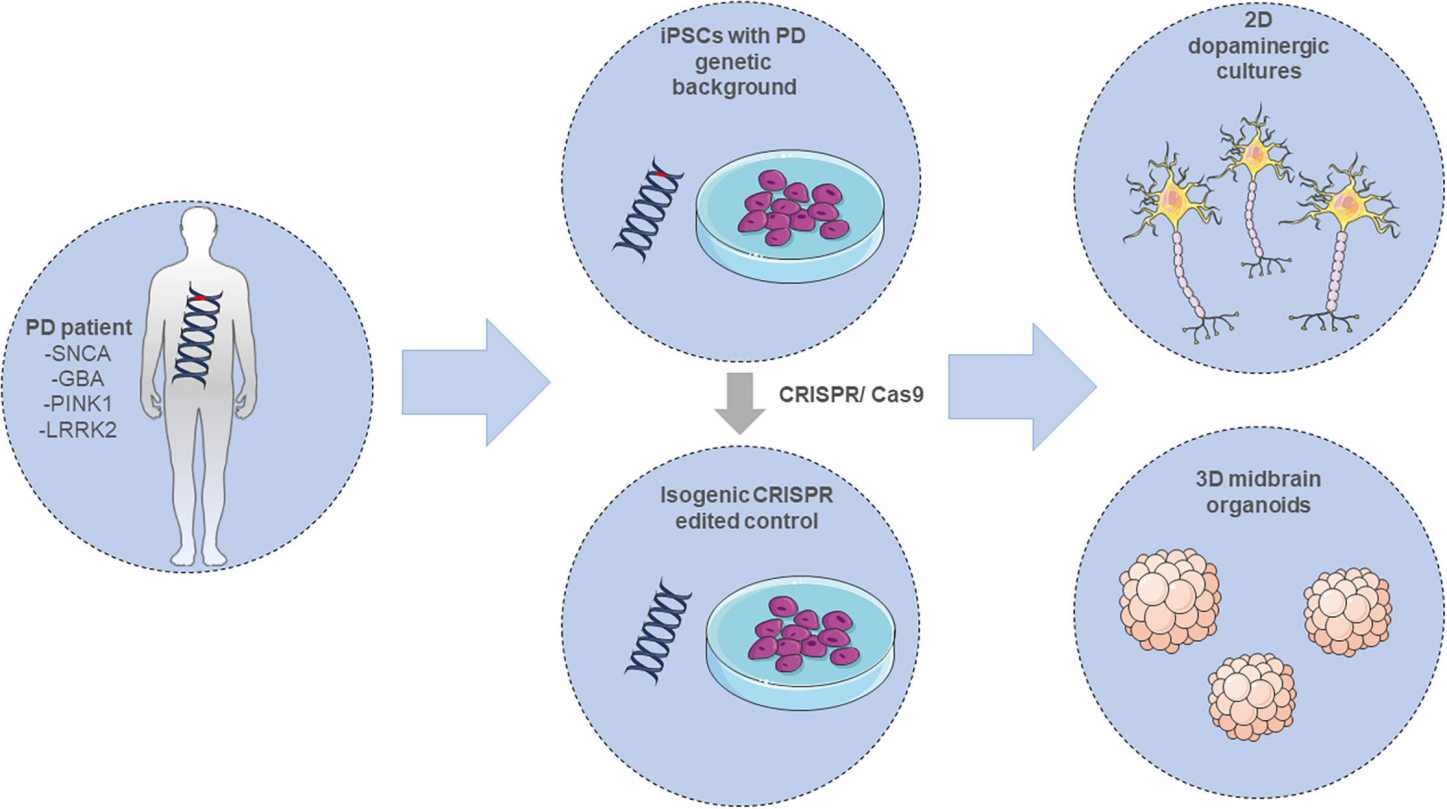
Modeling PD employing iPSC-derived *in vitro* systems. Adult differentiated cells, either from PD patients carrying a mutation in the *SNCA, GBA, PINK1*, or *LRRK2* gene amongst others, or CRISPR-edited isogenic controls, are reprogrammed into induced pluripotent stem cells (iPSCs). These cells can be further differentiated into 2D or 3D neuronal *in vitro* cultures for investigating PD pathology. The illustration was created using images from https://smart.servier.com/.

Recently, a new era of iPSC-modeling was initiated by the possibility of generating three-dimensional (3D), self-organizing cerebral organoids (134). These *in vitro* “mini-brains” are originally generated from iPSCs that first organize into EBs and later get embedded into an extracellular matrix hydrogel (e.g., matrigel) and differentiate toward the neuroectodermal lineage. The organoids recapitulate many key structures of the human brain, like the cortical “inside-out” architecture, including a lumen, a subventricular zone (SVZ), a cortical plate (CP) and choroid plexus-like structures. These 3D *in vitro* brain-like structures consist of a variety of cell types, showing also the ones found in the developing human brain. The organoid cellular composition contains neural progenitors, glial cells, intermediate progenitors as well as deep- and upper-layer neurons (135). Further developments on the organoid technology have resulted in region-specific organoids, recapitulating neuroanatomical structural features of various human brain regions including forebrain (136, 137), pallium and subpallium (138), choroid plexus (139), cerebellum (140), retina (141), hypothalamus (142), and finally midbrain (143–145). Especially, the later ones are of high relevance in the PD research field and can constitute a promising novel *in vitro* tool providing new insights into the early neurodevelopmental stages of the disorder (Figure 3).

Different protocols have been developed for the creation of homogeneous midbrain-like organoids (MOs). Tieng et al. described a “neurosphere” mediated method, where first the iPSCs form EBs that then get exposed to a FP-inducing medium, containing LDN193189, SB431542, SHH, FGF8, purmorphamine, and CHIR99021 and finally to a neural maturation medium. This protocol yields organoids that contain around 60% of DA neurons, in around 3 weeks (146). Alternative protocols similarly follow the EB formation method and e.g., employ the exposure of the cells to several morphogens, like Wnt activators, dual SMAD inhibitors and SHH activators (143, 147, 148). Similarly, to Tieng et al. these protocols also report high proportion of DA neurons, >60%, together with functional glial cells, astrocytes and oligodendrocytes. All protocols also result in electrophysiologically active neurons, however the protocol developed by Jo et al. was the only one that reported the identification of SNpc DA neuron-like electrophysiological patterns (149). Interestingly, Kwak et al. observed DA specific cell death after the treatment with 1-methyl-4-phenyl-1,2,3,6-tetrahydropyridine, indicating this 3D system as a relevant PD model (148). Even though there is a variety of protocols for deriving midbrain organoids, the usage for PD modeling is still in its infancy.

### iPSC-Derived Neuronal Models to Mimic PD

#### Copy Number Variations of *SNCA*

With the establishment of DA neuronal differentiation protocols, researchers started to employ them in order to generate DA neurons with PD-related genetic backgrounds. Some of the first studies focused on the generation of DA neurons from iPSCs carrying a *SNCA* triplication (*SNCA*-tri) genetic variant. These studies revealed further insight into the occurrence of PD pathological hallmark features, like α-syn aggregation (150–157), mitochondrial dysfunction and elevated oxidative stress (150, 156, 158), as well as ER stress (154, 159).

Byers et al. report that PD midbrain DA neurons overexpressed oxidative stress related markers, like heme oxygenase 2 (HMOX2), when compared to healthy controls. The disease related neurons appeared to be more vulnerable to oxidative stress and they showed increased cell death (150). Mitochondrial dysfunction accompanied by increased oxidative stress was also observed by Zambon et al. The group reported decreased cellular respiration accompanied by abnormalities in the mitochondrial membrane potential that could be induced by the reduced phosphorylation of Dynamin-related protein 1 (DRP1^Ser616^), a GTPase important for the mitochondrial morphology (156). Along the same lines, Ludtmann et al. observed mitochondrial accumulation of α-syn oligomers. The oligomers selectively oxidized mitochondrial proteins, e.g., the ATP synthase among others, an event that induced the early opening of the mitochondrial permeability transition pore (MPTP) that eventually led to mitochondrial swelling and increased cell death. These pathological effects were reversed after the inhibition of MPTP with the use of cyclosporin A (158).

It is considered that lysosomal function plays a crucial role in PD pathology. There is substantial evidence of an association between synucleinopathies and Gaucher disease, a lysosomal storage disorder (LSD) that is characterized by mutations in the glucocerebrosidase (GBA) gene (160, 161). In fact, mutations in the GBA gene in mice have been reported to result in accumulation of α-syn and finally lead to neurotoxicity. Moreover, the aggregations result in further inhibition of the normal GBA lysosomal function (161). This connection indicates that the impairment in the physiological lysosomal function reinforces the accumulation of α-syn, and thus contributes to the PD pathology (162). Mazzulli et al. confirmed in an *in vitro* cell culture model that iPSC-derived midbrain DA neurons, carrying a *SNCA* triplication or an A53T point mutation, exhibited reduced lysosomal function leading to elevated α-syn accumulation. Furthermore, a disruption in the subcellular protein sorting of the Rab1a protein, a GTPase with important ER-Golgi vesicular transport function, was reported, resulting in reduced hydrolase trafficking and eventually fragmentation of the Golgi apparatus. These pathological findings could be reversed with the overexpression of Rab1a in DA neurons (154). In a later study the same group showed the restoration of the lysosomal activity and the subsequent reduction of α-syn accumulation using a small molecule modulating the function of the lysosomal β-glucocerebrosidase enzyme (GCase) (163).

In the nuclei of *SNCA*-tri neurons perturbations of the normal cellular morphology were reported. Impairments of the nuclear envelope in PD neurons were shown along with abnormalities in the nuclear protein transport after the interaction of α-syn with Ras-related nuclear protein (RAN) (164). Similar findings were also described by Vasquez et al. in a study that employed *SNCA*-tri neural progenitor cells (NPCs). The group confirmed the nuclear localization of α-syn and its association with chromatin. The aggregates induced DNA damage and ultimately, increased cell death was reported (165).

Following the general developmental impairment that characterize pathological features of PD, studies reported a maturation delay of diseased neurons after comparing their transcriptomic profile with either healthy controls or primary midbrain DA neurons. Oliveira et al. reported the decreased expression of many genes that are implicated in neuronal differentiation processes (152). A year later, Xia et al. confirmed this finding. Among others, they described the downregulation of 37 genes in the PD midbrain DA neuronal cohort. The list included genes that are important for the DA neuronal identity, like TH and LMX1B, genes that are involved in neuronal maturation, as well as genes that are considered as risk genes for neurological pathologies (166). Further studies described developmental and morphological neuronal abnormalities, such as aberrant neurite morphologies showing reduced lengths and irregularities of the membrane integrity. Furthermore, Lin et al. reported that the *SNCA*-tri neurons exhibited decreased firing rates and complete absence of synchronized neuronal firing, indicating a decline in normal neuronal activity and connectivity (153). *SNCA*-tri neurons with impaired neurites were also reported by a study of Siddu et al. The number of healthy neurites was ameliorated after treatment with cysteamine that additionally reduced the levels of the α-syn aggregates (157). Cysteamine is an aminothiol derivative that is physiologically produced from the degradation of coenzyme A (167, 168). Notably, cysteamine exhibits a protective role against DNA damage when induced by irradiation since it acts as a reactive oxygen species (ROS) scavenger (169). Moreover, the compound has been used in multiple Huntington's disease (HD) preclinical studies with promising results, including prolonged mice survival (170–172). Siddu et al., after having additional encouraging results on mouse studies, proposed that cysteamine could be considered as a potential future drug candidate for PD (157).

Since PD is an age-related neurodegenerative disorder, mimicking the aging aspect plays a crucial role in the disease modeling. One of the limitations of the iPSC models is the rejuvenation of the cells during their reprogramming. In an auspicious study led by Chiba-Falek, the scientists bypassed this drawback with the generation of aged NPCs without the ectopic expression of genes or the use of toxins. In detail, the aged NPCs were derived after extensive passaging, 14–16 times. The aging phenotype was confirmed by assessing the expression of heterochromatin markers (e.g., histone 3 lysine 9 trimethylation, H3K9me3), by evaluating the nuclear membrane structure (e.g., stainings with lamin A/C), DNA damage (with cellular stainings of heterochromatin protein 1 γ, HP1γ) and the evaluation of the global methylation. The aged NPCs expressed age-related markers and gave rise to midbrain DA neurons that attained the aged phenotype. Notably, even the young *SNCA*-tri neurons, derived from young NPCs, showed increased aging-related markers when compared to control neurons (173).

The transfer of α-syn aggregates from *SNCA*-tri neurons to neighboring wild type neurons has been reported by Reyes et al., indicating a potential proof of the prion hypothesis for PD pathogenesis (174). Prions are defined by their “protein-only” composition that is—though devoid of nucleic acids—able to carry infectivity by direct protein-protein contact (175). This co-culture system can also be used as a small molecule screening system (174).

Besides iPSC models that are based on the triplication of the α-syn gene, some others carry *SNCA* duplications. This genetic background also led to the generation of neurons that exhibited hallmark pathological PD features, in a lesser extent compared to the triplications, like α-syn aggregations that specifically accumulated in midbrain DA neurons, mitochondrial dysfunction and energy deficits, synaptic loss, ROS accumulation, protein nitration and ultimately increased cell death (155, 176).

Taken together, multiplication of the *SNCA* locus, either triplication or duplication, appears adequate to initiate PD pathology (177, 178). The *SNCA* copy number variation (CNV) leads to PD symptom manifestation, like α-syn aggregation, lysosomal and mitochondrial impairments and accelerated neuronal cell death. Therefore, the *SNCA* multiplication iPSC-derived neuronal models appear to be useful in mimicking PD pathology. They offer valuable, new insights into the pathological pathways of PD initiation and progression and can also be used as drug screening platforms. The relevant studies and their main findings are summarized in Table 1.

**Table 1:** Summary on 2D models using iPSC and iPSC-generated neuronal cells having a PD-*SNCA*-related genetic background.

**Gene**	**Mutation**	**Cell type**	**Main findings**
*SNCA*	Triplication	Midbrain DA neurons	• α-syn aggregations (150–157) • Neuron to neuron α-syn transfer (174) • Mitochondrial impairment, oxidative stress, ER stress (150, 153, 154, 156, 158) • Lipid regulation impairment (156) • Neuronal maturation delay (152, 166) • Nuclear envelope perturbations (164) • Lysosomal dysfunction (154) • Increased cell death (150)
*SNCA*	Triplication	Aged NPCs, DA and cholinergic neurons	• α-syn aggregations (173) • Extensive passaging of NPCs lead to aged neurons (173) • *SNCA*-Tri neurons derived from young NPCs show accelerated nuclear aging (173)
*SNCA*	Triplication	NPCs	• α-syn aggregations (165) • α-syn nuclear localization (165) • DNA damage (165)
*SNCA*	Duplication	Midbrain DA neurons	• α-syn aggregations (155, 176) • Mitochondrial impairment (155, 176) • Energy deficits (155) • Increased cell death (176)
*SNCA*	A53T	Midbrain DA neurons	• α-syn aggregations (156, 179, 180) • Mitochondrial impairment, oxidative stress (155, 156, 180–182) • nitrosative and ER stress (156, 182, 183) • Synaptic impairment (179) • Lipid regulation impairment (156)
*SNCA*	A53T	Midbrain DA neurons	• Generation of the computational tool *TransposeNet* (184) • Generation of a genome-scale “humanized” map of α-syn toxicity (184)
*SNCA*	A53T	iPSCs	• Correction of the A53T mutation with BAC targeting vectors (185) • Generation of PD iPS lines by ZNF editing (186)
*SNCA*	A53T	DA NPCs	• α-syn fibril uptake (187) • α-syn transfer (187)
*SNCA*	E46K	Midbrain DA neurons	• α-syn aggregations (155) • Mitochondrial impairment (155) • Reduced axonal density (155) • Synaptic impairment (155)

#### *SNCA* Point Mutations

PD is also associated with various point mutations in the *SNCA* gene, with A53T and E46K being the most extensively studied ones. Like for CNVs iPSC-derived neuronal models based on *SNCA* point mutations do also result in α-syn aggregation (155, 156, 179, 180). Fernandes et al. showed in A53T-mutant midbrain DA neurons dysregulated expression of genes, related to chromatin organization and histone modifications. They also demonstrated a repression of oxidative phosphorylation and a reduction in the cholesterol biosynthesis (182). Studies of multiple research groups also report ER and mitochondrial stress linked to these point mutations. Interestingly, Ryan et al. described impaired and fragmented mitochondria that were also observed in control neurons after co-culture with *SNCA*-A53T neurons, indicating the transfer of α-syn fibrils of the pathogenic neurons to the isogenic control. This cell-to-cell transfer was inhibited by a monoclonal antibody directed against α-syn. Moreover, the same study identified that treatment with cardiolipin, a group of phospholipids that account for around 20% of the inner mitochondria membrane phospholipid mass, reduces α-syn accumulation at the mitochondrial outer membrane (180). A study on A53T-NPCs revealed lysosomal storage of α-syn aggregations and cell-to-cell transfer. The aggregates were found to be accumulated in tunneling nanotubule (TNT)-like structures (187). Kouroupi et al. described synaptic impairments that were reversed after treatment with a *de novo* designed small molecule, inhibiting the accumulation of α-syn (179). Khurana et al. developed an *in silico* tool, the so-called *TransposeNet*, a computational method that can interchange molecular interactions across species. It allows the generation of genome-scale maps of α-syn toxicity in yeast that then could reveal links to PD-relevant genes and to candidates targeting α-syn toxicity in humans. These then can be tested *in vitro* e.g., in patient-derived neurons (184).

PD is considered a complex disorder, influenced by a combination of both genetic and environmental factors. Recently, an intriguing study performed by Stykel et al. proved a genetic-environmental interaction by inhibiting the anterograde mitochondrial transport in *SNCA*-A53T harboring DA neurons. When exposed to the agrochemicals rotenone, or paraquat and manet, the mutated DA neurons showed a change in the nitration of α-tubulin (α-Tub), which led to altered microtubule architecture and eventually to the arrest of the anterograde mitochondrial transport toward the axon terminal (181). In contrast, the isogenic control neurons showed only minor effects to the exposure. As shown before, defects in mitochondrial transport are critical for the neuronal physiology (188). Therefore, Stykel et al. showed an additive effect of the environmental toxins, paraquat, manet and rotenone with the PD susceptible genetic background, that could lead to an earlier PD onset. The use of the nitric oxide biosynthesis inhibitor, Nω-nitro-L-arginine methyl ester, reversed these effects (181).

In conclusion, like the *SNCA* CNV models, point mutations also lead to the manifestation of PD hallmarks, ranging from α-syn aggregation to ER and mitochondrial stress. Though, a particular characteristic of the point mutation models is the wide variety of pathological observations. The relevant studies and their main findings are summarized in Table 1.

Notably, all 2D DA midbrain models involving *SNCA* triplication, duplication, or point mutations (A53T and E46K) reproduced the ability to aggregate α-syn (150– 153, 155–157, 163, 176, 179, 180), which further led to mitochondrial impairment (150, 155, 156, 158, 176). Furthermore, DA midbrain neuronal cells showing the *SNCA* triplication and the point mutation A53T genetic background revealed oxidative stress, ER stress and lipid dysregulation (150, 156, 158, 182), whereas increased cell death was found in the *SNCA* triplication and duplication DA midbrain neuronal cell lines (150, 176). Neuronal maturation delay in iPSC-derived DA midbrain neurons was shown in two separate studies using the *SNCA* triplication background (152, 166). Additional features or impairments were found in the different models, yet most of them need further verification.

The generation of 3D iPSC derived *in vitro* models allowed to bypass many of the limitations of the 2D counterpart. More specifically, the organoid models recapitulate more precisely the *in vivo* physiology and development, features that are not possible to be modeled in a simpler 2D cell culture model. Furthermore, organoids offer a higher cellular complexity, they allow complex cell-to-cell interactions and recapitulate the spatial architectural features of human development (189, 190). Nevertheless, 2D models are still useful and well-established providing easier and less complicated data interpretation.

Given the recent development of the 3D neuronal organoid technology, not many studies employed organoids derived from iPSC lines expressing *SNCA* mutations, so far. Based on previous studies Jo et al. generated *SNCA*-triplication and *GBA1* knock-out midbrain organoids that successfully exhibited α-syn aggregation, LB generation and DA neuronal loss (149). A very recent study focuses on the establishment of *SNCA*-triplication midbrain organoids employing CRISPR/Cas9 edited isogenic controls. This study also stated the neuronal and glial accumulation of α-syn aggregates that interestingly increased in organoids of higher age, recapitulating the physiological age-dependency of PD pathology (191). Smits et al. focused on the establishment of *LRRK2*-G2019S midbrain organoids. These PD midbrain organoids exhibit disease-related phenotypes, like impaired neuronal complexity and interestingly, they showed a decreased number of DA neurons, even though there was an excess of FOXA2+ progenitors, indicating a developmental impairment in the DA neuronal differentiation (147).

Aside from the *SNCA* gene variants, PD GWAS studies identified a plethora of disease-associated risk variants that still need further functional studies, focusing on the underlying pathological mechanisms (192). Therefore, the generation of different disease-relevant 3D models, but also 2D models, recapitulating the genetic background of PD, will be of great significance in order to get more insights into PD initiation and progression.

## Discussion and Conclusion

PD is a neurodegenerative disorder affecting a substantial fraction of the global population. The disease is both, devastating for the patients' life quality, and also a big burden for the global healthcare system. Due to the restricted access to PD-affected human brain tissue there is a fundamental need for reliable *in vivo* and *in vitro* models. The current *in vivo* models employ a wide range of animals including rodents, non-mammalian species and non-human primates (NHP). Transgenic animal models, especially mice, have been extensively used for studying PD, since they do not only enable insights into the pathogenesis of PD, but also can be employed for the identification and validation of new potential therapeutics (193, 194). Reported mouse models include transgenic α-syn models and transgenic animals with mutations in the *LRRK, PINK1* and *DJ-1* gene (194). *In vitro* PD cellular models include the use of various cells lines, both primary and immortalized. Among the most widely used lines for PD modeling are the neuroblastoma immortalized cell line SH-SY5Y (195), the Lund human mesencephalic immortalized cell line (LUHMES) (196), the rat N27 immortalized mesencephalic cells and primary DA cells (197). The dopamine synthesis and metabolism machinery of these lines turns them into suitable PD models.

Here, we highlighted the importance of developing complementary *in vitro* iPSC-derived neuronal models for studying particularly the involvement of α-syn in PD pathology. These models can offer valuable insights into the pathology mechanisms, but they could also serve as platforms for drug screening. The iPSC technology allows the generation of models of human origin, carrying the patient's genetic background, which makes them a useful tool for getting insights into the pathological disease mechanisms. Furthermore, these systems are readily available, for instance through various biobanks [among others the Corriel (https://www.coriell.org/1/Browse/Biobanks) and the European Bank for induced pluripotent Stem Cells (EBiSC)], as well as through various commercial sources, and well-established for disease modeling, as it is apparent from the numerous studies employing iPSCs for this purpose (120).

PD is a complex multifactorial disorder. iPSCs can be employed for addressing several pathophysiological mechanisms and life-style parameters that are implicated in the disorder. For example, there are studies deciphering the implication of inflammation in PD, employing iPSC-derived macrophages (198) and microglial cells (199). The influence of metabolism and diet were addressed in metabolomic studies (200–202) and studies that control the nutrient composition of the culture medium (203). However, other aspects, like the effect of exercise in disease progression are hard to be modeled in an *in vitro* system and therefore an *in vivo* mouse model would be the appropriate system for this scientific question.

However, though iPSC are powerful tools in generating patient-specific diseased cells, they exhibit several limitations. Genomic instability and epigenetic aberrations potentially induced by reprogramming need to be carefully monitored (204, 205). Moreover, iPSC reprogramming involves a global epigenetic reset representing an undesired cell rejuvenation process if it comes to study age-related disorders (206). Furthermore, for a clinical application of iPSC-derived cells the potential tumorigenicity and immunogenicity need to be addressed (207–209). Finally, since iPSCs carry the patient's genetic background, special attention needs to be dedicated into the proper handling of the personal, genomic data (210, 211).

To sum up, iPSC-derived *SNCA* neuronal models are either focusing on *SNCA* gene multiplications or mimic the *SNCA* disease-related point mutations. Even though these models partly enable the recapitulation of PD hallmark pathological features, like α-syn aggregation, mitochondrial stress, lysosomal dysfunction, ER stress, DNA damage and accelerated cell death, none of them managed to demonstrate the combination of all these pathological characteristics. Additionally, some of the studies confirmed the transfer of α-syn fibrils from *SNCA*-tri neurons to healthy controls. Despite the lack of an ultimate iPSC-derived PD model that demonstrates holistically the pathological PD hallmarks, these studies using iPSC-derived PD models identified significant findings regarding the pathological pathway involved in PD initiation and progression.

However, iPSC-derived PD models are poor in modeling aging, due to the rejuvenation of the cells during the reprogramming (206, 212, 213). In fact, PD is an age-related pathology that manifests mostly in patients with higher age. Some studies addressed this issue by artificial aging, e.g., by extensive NPC passaging initiating the expression of age-related markers and later differentiating them into neurons (173). The iPSC rejuvenation could also be bypassed by transdifferentiation, also known as direct conversion, a technique that differentiates the adult cells into the cell type of interest, without passing through the reprogrammed state. This way, the cells retain their age and could provide a better suited model for studying late onset disorders (214). In the context of PD, adult dermal fibroblasts have been directly converted into DA neurons through the overexpression of a cocktail of transcriptional factors (215, 216). Another study, has also generated DA neurons by directly converting astrocytes (217).

Even though, direct conversion into DA neurons is a great tool, the generated neuronal population is post-mitotic. Therefore, there are several concerns about the efficacy and the survival of the neuronal populations in cases of transplantation for therapeutic purposes. Another promising alternative is the use of NPCs. NPCs are multipotent progenitor cells that can give rise to neurons, oligodendrocytes and astrocytes (218–221) and novel reprogramming paradigms allow direct conversion of patient-specific cells into NPCs (221–223). NPCs combine a series of interesting features, like the partial maintenance of their aging signatures, such as induced neurons. They are also safer for cell therapy since they are not tumorigenic, in contrast to iPSCs. More importantly they are expandable, making them ultimately a promising therapeutical approach.

The exogenous administration of α-syn pre-formed fibrils is another alternative approach in PD modeling. There is extensive use of this approach in animal models, including mice and non-human primates (224). Although, many insights were gained from these *in vivo* studies, the animal models do not recapitulate the full spectrum of the human PD pathology. Therefore, translating the results of the neuronal reactions to extracellular human α-syn from animals to humans is a difficult task. The same approach has recently been used in an iPSC-derived neuronal system. The study established 2D cortical neuronal networks using microfluidic devices. The healthy neurons of the one side of the microfluidic device were incubated with α-syn fibrils and the researchers assessed the uptake, the intracellular fibril transfer, and the cell-to-cell fibril transfer. They reported a prion-like α-syn fibril transfer between the two neuronal populations. Moreover, the α-syn aggregates led progressively to the appearance of PD pathological features (225).

Conclusively, it is apparent that there are exciting new developments regarding the PD research field, both in basic research and in the applied clinical field. Many emerging new technologies, e.g., midbrain organoids and multi-omics data, promise to give invaluable new insights to the research community. Based on the literature though, it is evident that there is a need for additional studies employing these new models. The upcoming years will bring a great influx of exciting new data that hopefully will lead to better understanding of the PD pathology helping to generate new treatment options that will ameliorate the patients' lives or even stop the progression of the disease.

## Author Contributions

AS wrote the initial draft of this review. LF and FE conceptualized the review and reviewed the final manuscript. All authors were involved in revising and editing the manuscript, and they all read and approved the final version of the manuscript.

## Funding

This work was supported by the Austrian Science Fund (FWF, I 4791-B and SFB F7810), the German Research Foundation (DFG; ED 79/4-1) to FE, and by the European Union's Horizon 2020 Marie Sklodowska-Curie research grant No. 847681 (ARDRE).

## Conflict of Interest

The authors declare that the research was conducted in the absence of any commercial or financial relationships that could be construed as a potential conflict of interest.

## Publisher's Note

All claims expressed in this article are solely those of the authors and do not necessarily represent those of their affiliated organizations, or those of the publisher, the editors and the reviewers. Any product that may be evaluated in this article, or claim that may be made by its manufacturer, is not guaranteed or endorsed by the publisher.
